# Micro-anatomical quantitative optical imaging: toward automated assessment of breast tissues

**DOI:** 10.1186/s13058-015-0617-9

**Published:** 2015-08-20

**Authors:** Jessica L. Dobbs, Jenna L. Mueller, Savitri Krishnamurthy, Dongsuk Shin, Henry Kuerer, Wei Yang, Nirmala Ramanujam, Rebecca Richards-Kortum

**Affiliations:** Department of Bioengineering, Rice University, 6500 Main Street, BRC 502, Houston, TX 77030 USA; Department of Biomedical Engineering, Duke University, 101 Science Drive, Room 136 Hudson Hall, Box 90281, Durham, NC 27708 USA; Department of Pathology, University of Texas MD Anderson Cancer Center, 1515 Holcombe Boulevard, Unit 1350, Houston, TX 77030 USA; Department of Surgical Oncology, University of Texas MD Anderson Cancer Center, 1515 Holcombe Boulevard, Unit 0444, Houston, TX 77030 USA; Department of Diagnostic Radiology, University of Texas MD Anderson Cancer Center, 1515 Holcombe Boulevard, Unit 1350, Houston, TX 77030 USA

## Abstract

**Introduction:**

Pathologists currently diagnose breast lesions through histologic assessment, which requires fixation and tissue preparation. The diagnostic criteria used to classify breast lesions are qualitative and subjective, and inter-observer discordance has been shown to be a significant challenge in the diagnosis of selected breast lesions, particularly for borderline proliferative lesions. Thus, there is an opportunity to develop tools to rapidly visualize and quantitatively interpret breast tissue morphology for a variety of clinical applications.

**Methods:**

Toward this end, we acquired images of freshly excised breast tissue specimens from a total of 34 patients using confocal fluorescence microscopy and proflavine as a topical stain. We developed computerized algorithms to segment and quantify nuclear and ductal parameters that characterize breast architectural features. A total of 33 parameters were evaluated and used as input to develop a decision tree model to classify benign and malignant breast tissue. Benign features were classified in tissue specimens acquired from 30 patients and malignant features were classified in specimens from 22 patients.

**Results:**

The decision tree model that achieved the highest accuracy for distinguishing between benign and malignant breast features used the following parameters: standard deviation of inter-nuclear distance and number of duct lumens. The model achieved 81 % sensitivity and 93 % specificity, corresponding to an area under the curve of 0.93 and an overall accuracy of 90 %. The model classified IDC and DCIS with 92 % and 96 % accuracy, respectively. The cross-validated model achieved 75 % sensitivity and 93 % specificity and an overall accuracy of 88 %.

**Conclusions:**

These results suggest that proflavine staining and confocal fluorescence microscopy combined with image analysis strategies to segment morphological features could potentially be used to quantitatively diagnose freshly obtained breast tissue at the point of care without the need for tissue preparation.

**Electronic supplementary material:**

The online version of this article (doi:10.1186/s13058-015-0617-9) contains supplementary material, which is available to authorized users.

## Introduction

Breast cancer diagnosis is an intricate process, which requires tissue procurement, rigorous tissue preparation and histologic assessment whether it is in the context of core needle biopsy diagnosis or surgical excision. Fixed tissue samples are processed after harvesting and are evaluated for presence and type of malignant breast tissue based on standardized histologic criteria [1–4], which employ cytological and qualitative architectural features. Breast tumors that are diagnosed as malignant in nature are graded using different types of grading systems to categorize the tumors into groups to reflect their biology of progression. One of the most widely used grading systems was developed by Bloom and Richardson in 1957, which used only qualitative criteria to evaluate breast lesions [2]. In 1991, Elston and Ellis published the Nottingham modification to the Bloom and Richardson grading system, which incorporated semi-quantitative criteria to evaluate tubule formation, nuclear pleomorphism, and mitotic count [4]. Extensive research has been done to evaluate the rate of inter- and intra-observer discordance using these grading systems for histologic assessment of fixed breast tissue. While some studies have shown that inter-observer agreement is high in the majority of cases [5], other studies have shown that subjective criteria can lead to inter-observer variation for margin assessment and poor reproducibility in evaluation of borderline and in situ lesions [6–11]. The availability of techniques that use quantitative criteria that can be applied without subjecting the tissue to processing can overcome the subjectivity of interpretation and may reduce the inter- and intra-observer variability in the histological evaluation of breast tissue [12]. Such techniques could also be potentially useful in settings lacking the human resources and equipment necessary to perform standard histologic assessment, which can be a challenge in many parts of the world [13].

In order to characterize quantitative criteria to classify breast architecture, several studies have described segmentation algorithms based on nuclear [14–18] and ductal [19–21] morphometry in images of fixed tissue stained with hematoxylin and eosin (H&E) staining. Additionally, some recent studies evaluated nuclear morphometric parameters using wide-field fluorescence microscopy [22] and micro-optical computed tomography [23] to acquire images of breast tissue. Specifically, wide-field fluorescence microscopy combined with watershed segmentation to quantify nuclei found that area fraction could distinguish between tumor and normal regions in excised rat mammary tissue with 97 % accuracy [22]. Micro-optical computed tomography and nuclear morphometry was used to compare variations between human breast cell lines and found that nuclear volumes increased from normal to metastatic breast cells and that nuclei of abnormal cells contained more nucleoli [23].

The idea of establishing quantitative criteria on fixed tissue can be taken one step further to be applied directly to intact specimens using other imaging modalities, which can obviate the need for extensive tissue processing. Several studies have already described the feasibility of imaging breast tissue with confocal microscopy in a clinical setting, [24–29]. Schiffhauer and colleagues showed that confocal reflectance microscopy could be used to image benign and malignant breast features and provide visual similarity to H&E micrographs [26]. Abeytunge and colleagues demonstrated that confocal fluorescence microscopy can be used to rapidly acquire images of fresh tissue specimens between 1 and 2.5 cm^2^ in size [29]. Our group recently showed that confocal fluorescence microscopy yields images with sufficient detail to identify benign and malignant breast architecture in freshly excised tissue [24]. In another recent study, we demonstrated that confocal fluorescence images can be used to estimate percent tumor cellularity in core needle biopsy specimens and can indicate the adequacy of procured tissue for diagnosis and ancillary molecular and immunophenotypic studies [25].

The goal of this work is to combine both quantitative image processing techniques with optical microscopy of intact breast tissue specimens for interpretation of breast tissue at the point of care. The benefits of this approach are minimal tissue processing, rapid diagnosis, and quantitative criteria that could potentially reduce the subjectivity with intra- and inter-observer variation in the evaluation of breast histology. In this study, we combine clinical confocal microscopy with a computerized image processing algorithm to quantify both nuclear and ductal morphology of breast tissue; we develop an algorithm using these parameters to classify breast tissue as benign - negative for tumor - or malignant - tumor tissue present. Although previous studies have described evaluation of breast architecture in histologic images [14–21], these studies only considered either nuclear or ductal parameters. We show that combining both yields improved diagnostic performance, particularly in the diagnosis of invasive ductal cancer (IDC) and ductal carcinoma in situ (DCIS) The nuclear and ductal parameters described in this study could potentially be used for objective categorization of breast lesions.

## Methods

### Breast tissue acquisition and preparation

Fresh human breast tissue specimens from 34 patients were acquired through a protocol approved by The University of Texas MD Anderson Cancer Center and Rice University Institutional Review Boards, and each participant gave written informed consent. Fresh breast tissue was acquired from patients undergoing surgery to excise a clinically abnormal lesion. The procedure for tissue preparation has been described previously [24]. In brief, two tissue specimens - one grossly abnormal and one grossly normal in appearance were acquired from each patient for image acquisition and evaluation; each specimen measured approximately 15 × 15 mm^2^ in size, with thickness varying from 2 to 7 mm. Within 30 min of surgical excision, breast tissue specimens were stained for 1 min in a solution of 0.01 % proflavine in 1X phosphate-buffered saline (PBS). Proflavine is a nuclear contrast agent [30, 31], which has been used to stain breast tissue, oral mucosa, Barrett’s esophagus, cervical tissue, and sarcoma in previous studies [24, 25, 32–37]. Following topical application of proflavine, specimens were washed with 1X PBS and then immediately imaged.

### Image acquisition and evaluation

Confocal fluorescence images were acquired from multiple sites within each specimen using a multi-wavelength scanning confocal microscope (Vivascope 2500®, Caliber Imaging and Diagnostics Inc., Andover, MA, USA) as described previously [24, 25, 38]. Following topical application of proflavine and the PBS wash, each tissue specimen was positioned on the microscope stage and imaged using 2.1 ± 0.4 mW power at 488 nm laser excitation, and the fluorescence was detected in a band pass of 550 ± 44 nm with a 30× water immersion lens. At these settings, the lateral and axial resolution was 1.0 μm and 5.0 μm, respectively, in the center of the 750 × 750 μm^2^ field of view. A 12 × 12 mm^2^ composite image was created for both sides of each tissue specimen. To create the composite image, images were acquired from contiguous sites in a grid pattern (maximum area 12.2 × 12.2 mm^2^) over the surface of the specimen at an approximate depth of 20 μm. Following image acquisition, specimens were kept moist in 1X PBS and were submitted for routine histologic preparation and fixation. Samples were stained with H&E and fixed on microscope slides for histologic assessment.

A board-certified, breast pathologist (author S. Krishnamurthy) viewed composite confocal images and fixed tissue specimens stained with H&E using a conventional light microscope to identify sites that corresponded to the same approximate location in the specimen based on similar image morphology. Specifically we selected in-focus confocal microscope fields of view that contain representative examples of characteristic benign and malignant breast features. Thus, at each site, a corresponding pair of confocal and H&E images were available from a 750 × 750 μm^2^ field of view. At each site, the H&E images of fixed tissue specimens were used as a reference standard to identify breast architectural features that should be present in corresponding confocal images [1, 24]. Benign breast features identified in reference H&E images included adipose and fibrous tissue, lobules, non-hyperplastic ducts, and ductal hyperplasia. Malignant breast features identified in reference H&E images included: ductal carcinoma *in situ* (DCIS), invasive ductal carcinoma (IDC), and invasive lobular carcinoma (ILC). Benign breast features were identified in specimens acquired from 30 patients and malignant features were identified in specimens from 22 patients.

### Nuclear segmentation and connected components algorithms for identifying nuclei

**Fig. 1:**
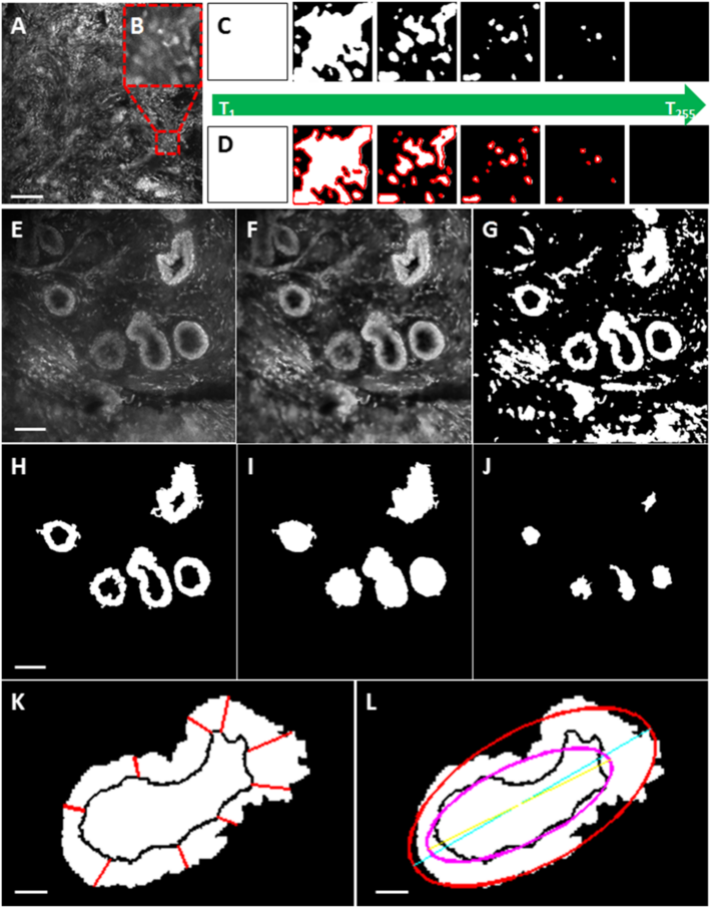
Algorithms for nuclear (**a**-**d**) and ductal (**e**-**l**) segmentation. Nuclear segmentation: **a**: Raw image acquired from confocal fluorescence microscope with 750 × 750 μm^2^ field of view. **b**: Region of interest selected in confocal fluorescence image with 75 × 75 μm^2^ field of view. **c**: The maximally stable extremal regions (MSER) algorithm applies thresholds from 0 to 255 to **b**. **d**: At each threshold, the MSER algorithm identifies nuclei as connected components and selects “maximally stable” components with the lowest size variation. Ductal segmentation: **e**: Raw image acquired from confocal fluorescence microscope with 750 × 750 μm^2^ field of view. **f**: Wiener lowpass filter and adaptive histogram equalization applied to **e**. **g**: The algorithm converts E to a binary image using an interactive threshold tool. **h**: Objects below range of nuclear area are removed and then user selects a region of interest (ROI) around ducts with an interactive polygon selection tool. **i**: The algorithm fills boundaries of ducts identified in (**h**) to segment the outer boundaries of the duct. **j**: The algorithm selects the complement of (**h**) to segment the inner boundaries of the duct (lumen). **k**: Duct wall width is measured by selecting the shortest distance from the outer to the inner duct boundaries (*red lines*). **l**: Ellipses are fitted to outer and inner duct boundaries. **e**-**j**: Scale bar is 100 μm. **k**, **l**: Scale bar is 25 μm

A technique called maximally stable extremal regions (MSER) was used to segment nuclei from confocal images of proflavine-stained breast tissue. MSER has been used previously in the image-processing community for automatic reconstruction of three-dimensional scenes; here we have adapted it to segment nuclei from high-resolution fluorescence confocal microscopy images [39]. MSER employs intensity thresholding; however, no global or optimal threshold is sought; rather all thresholds are tested and the stability of the isolated connected components (i.e. nuclei) is evaluated. All possible thresholds from 0 to 255 are applied to an image and the sets of connected components (as well as their area) are stored (Fig. 1a-d). This yields a data structure in which the area of each connected component is stored as a function of the intensity threshold. Finally, the intensity thresholds which correspond to local minima in the rate of change of the area function are selected as thresholds producing MSER.

In order to apply MSER to our images, five tuning parameters associated with MSER had to be selected. The first two parameters, which included the minimum area (MinArea) and maximum area (MaxArea) of the connected components, are related to the expected size of nuclei. These parameters were selected based on the biologically expected range of nuclear diameters. Specifically, other groups have found nuclear volume to range from approximately 200 to 1500 μm^3^, which corresponds to 7 to 14 μm in diameter [23]. Therefore, MaxArea was set to 500 pixels, which corresponds to 19 μm in diameter, which is larger than the expected nuclear size for our images. MinArea was set to 35 pixels, which corresponds to 5 μm in diameter, which is smaller than the expected nuclear size for our images. The next set of parameters is related to the intensity thresholds and includes maximum variation (MaxVariation), minimum diversity (MinDiversity), and Delta. MaxVariation is the maximum variation allowed within a region that corresponds to a potential nucleus. MinDiversity is employed if there are two nested maximally stable regions. Specifically, if the diversity between the two nested regions is less than MinDiversity, then the nested region is removed. Lastly, Delta is the stability threshold. The stability of a region is defined as the relative variation of the region area when the intensity is changed by Delta/2. These intensity parameters were systematically tuned through applying a range of values to representative images in order to select the best value for each parameter. Specifically, one input parameter was varied over a wide range while other input parameters were held constant. For each iteration, the area fraction (AF) from representative images of tumor and normal tissue was calculated and overlays of the features isolated with that particular setting were displayed. The values that led to the largest differences in AF between tumor and normal tissues, while isolating features that approximately corresponded to nuclei or nucleoli, were selected. An illustration of this tuning approach can be found in Figure S1 in Additional file 1. Specifically, MaxVariation was set equal to 2.5, MinDiversity to 0.5, and Delta to 6. These parameter values are in terms of relative intensity, which for our 8-bit images ranges from 0 to 255.

After nuclei were isolated with MSER, a connected components algorithm was applied in order to calculate parameters such as nuclear density and diameter. In the connected components algorithm, all touching or connected pixels are assumed to belong to the same cell nucleus. Parameters include nuclear density (the number of nuclei in a unit area), area fraction (the total nuclear area divided by the total area), minimum inter-nuclear distance (the distance from a nucleus center to the next closest nucleus center), and nuclear diameter (the length of the major axis of each nucleus). Nuclear density and AF represent scalar variables – only one value is returned for each image, while the minimum inter-nuclear distance (IND) and nuclear diameter represent vector variables – a value is calculated for each nucleus in the image. In order to consolidate the vector variables into a scalar value, several summary statistics were evaluated, including mean, median, mode, interquartile range, and standard deviation.

### Ductal segmentation algorithm and quantification of ductal parameters

An algorithm was developed to measure ductal parameters, which segments non-hyperplastic ducts, ductal hyperplasia, and DCIS lesions based on the intensity of proflavine staining (Fig. 1e-l). To reduce noise and increase image contrast, a Wiener lowpass filter was first applied (Fig. 1e) followed by contrast-limited adaptive histogram equalization (CLAHE, Fig. 1f). Images were converted from grayscale to binary using a user-defined threshold based on relative intensity. The mean threshold used to segment ducts was 107 ± 27 (range: 52–168) on a scale of 0 to 255 for 8-bit images (Fig. 1g). It was not possible to select a universal threshold, because in order to accurately segment ducts from surrounding tissue, it is necessary to isolate both nuclei in the duct walls and inter-nuclear space between them. The relative intensity of these features differed between images due to the variation in illumination power used for image acquisition and the variation in proflavine staining. Areas smaller than the upper threshold for cell nuclei (approximately equivalent to 280 μm^2^ or 500 pixels, with a diameter of 19 μm [40]) were removed to avoid segmenting individual nuclei outside of the duct walls. Individual ducts were manually segmented using a user-defined polygon selection tool to define architectural features corresponding to breast ducts (Fig. 1h). After application of the ductal segmentation algorithm, the binary confocal image showed the segmented duct walls (Fig. 1h) and the outer and inner boundaries (Fig. 1i, j) of the duct used to measure ductal parameters.

Following segmentation of ducts, a number of ductal parameters were measured based on the properties of the inner and outer duct boundaries. The outer boundary defines the outer edge of the duct wall and the inner boundary defines the inner edge of the duct wall; the lumen. The width of the duct wall was measured at every pixel on the outer edge of the duct wall. This was done by finding the shortest distance between every point on the outer boundary and the nearest point on the inner boundary (Fig. 1k). Duct wall width was measured for each non-hyperplastic duct, ductal hyperplasia, and DCIS lesion and the vector of values were summarized by calculating the mean, median, mode, interquartile range, and standard deviation. Other scalar parameters measured include the area of the duct wall, area of the lumen, area of an ellipse approximating the duct wall, area of an ellipse approximating the lumen, lengths of the major and minor axes for the duct and the lumen, solidity of the duct and the lumen, and eccentricity of the duct and the lumen (Fig. 1l).

### Statistical analysis and model building

Nuclear parameters were calculated for all sites (n = 259) and ductal parameters were calculated for all sites that contained ducts (n = 50), and the diagnostic performance of each image parameter was individually assessed by determining the classification accuracy. Two-class linear discriminant analysis was performed to classify malignant from benign breast architectural features based on each individual nuclear or ductal parameter; receiver operator characteristic (ROC) curves were constructed and area under the curve (AUC) was calculated for each ROC curve. Sensitivity and specificity values were determined at the optimal cutpoint. Parameters were sorted by accuracy for classification of neoplasia based on AUC values. Boxplots were created for the parameters with the highest AUCs. A Student *t* test for samples with unequal variances was used to identify statistically significant differences between mean parameter values measured in benign and malignant tissues. This analysis was performed to evaluate individual nuclear and ductal parameters to incorporate into a classification model.

Next we sought to develop a multivariate model to yield optimal separation between benign and malignant tissues. Toward that end, all 33 nuclear and ductal variables were used as input for a classification and regression tree (CART) function in Matlab. Decision trees were constructed using the automated Matlab function classregtree, which selects parameters and cutpoints that lead to the optimal classification of benign and malignant breast architectural features. Decision trees were pruned to prevent a single nuclear or ductal from being used at more than one node within the tree. Pruning was also performed to prevent the number of categories for classification of malignant breast features from exceeding three: the number of malignant tissue types (IDC, ILC, and DCIS). After construction, decision tree nodes were pruned by finding the next higher node whose decision point led to two categories, one with a majority of neoplastic sites, and one with a majority of benign sites. A custom leave-one-out cross-validation algorithm was also developed in order to calculate the cross-validated sensitivity and specificity. Specifically, 258 of the 259 data points were used to build a CART model, which contained the same two variables at the first and second decision points. Specifically the standard deviation of IND (StdIND) was the first decision point and the number of lumens was the second decision point. However, with each iteration of leave-one-out cross-validation, the cutoff value of StdIND could vary. The cutoff value associated with the number of lumens (number of lumens >1) was held constant because biologically normal ducts are expected to only contain a single lumen; therefore, this was considered to be the optimal and only logical cutoff value and therefore was held constant. Then the model was applied to the remaining data point, which was classified as either benign or malignant. This process was repeated for all 259 data points, and the calculated diagnosis for each image was compared to the known diagnosis in order to calculate sensitivity and specificity for the cross-validated model. The performance of the decision tree was characterized by computing sensitivity and specificity for classification of malignant breast architectural features. Additionally, sensitivity and specificity were calculated for each individual histologic type of malignant tissue in order to determine the relative classification accuracy for IDC, ILC, and DCIS sites. For example, in order to calculate sensitivity for IDC, true positives were defined as IDC sites that had been classified as malignant by the decision tree, and false negatives were defined as IDC sites that had been classified as benign. Specificity was calculated by defining true negatives as benign sites that were correctly classified in the decision tree and false positives were defined as benign sites that were incorrectly classified. An ROC curve was constructed for the decision tree model. All sites were sorted in order of ascending StdIND value and then sensitivity and specificity for classification of neoplasia were calculated at every StdIND value. The cutoff value for number of lumens was held constant at one lumen because biologically normal ducts are expected to only contain a single lumen. AUC was calculated based on the resulting ROC curve.

**Fig. 2:**
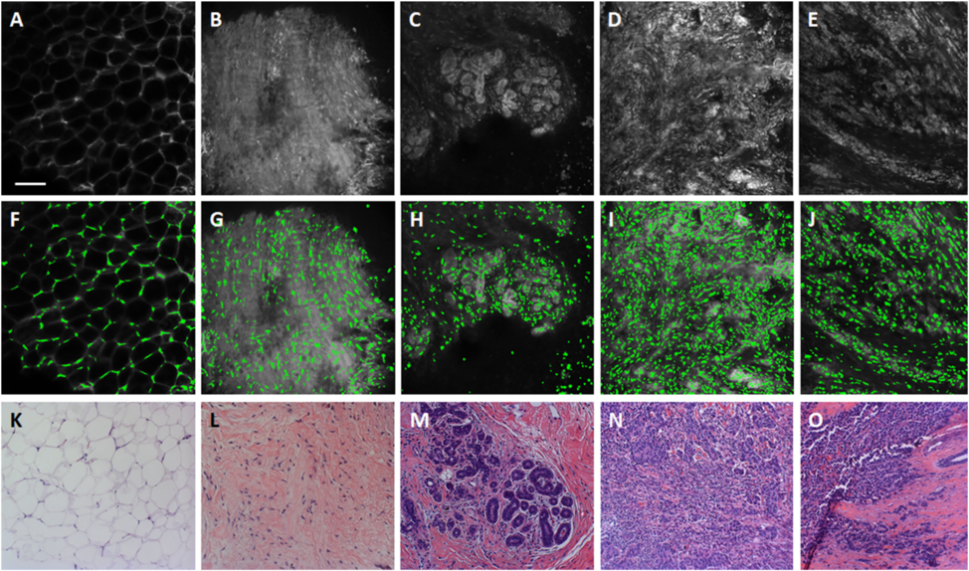
Representative raw confocal fluorescence images of adipose tissue, fibrous tissue, lobules, invasive ductal carcinoma, and invasive lobular carcinoma are shown in **a** through **e**, respectively. **f**-**J**: Nuclei segmented by identifying maximally stable extremal regions (MSER) are false colored green and overlaid onto the raw confocal fluorescence image. **k**-**o**: Histologic slides with H&E staining show similar histology to confocal images in **a**-**e**. Slides were prepared with the same specimens from which confocal images were acquired. Scale bar is 100 µm

## Results

A total of 259 sites from 34 patients were identified in composite confocal fluorescence images. A summary of patients, sites, and diagnoses are included in Table 1. In total there were 179 benign sites, which included adipose tissue, fibrous tissue, lobules, and benign ducts, and 80 malignant sites, which included DCIS, IDC, and ILC.

**Table 1:** Summary of patients from which tissue specimens were acquired, sites analyzed with segmentation algorithms, and histologic diagnoses for each site

Diagnosis	Patients	Sites
Benign	30	179
Adipose tissue	18	42
Fibrous tissue	14	31
Lobules	12	82
Non-hyperplastic ducts	6	20
Hyperplastic ducts	4	4
Malignant	22	80
Ductal carcinoma in situ (DCIS)	6	26
Invasive ductal carcinoma (IDC)	15	37
Invasive lobular carcinoma (ILC)	3	17
Total	34	259

Figure 2 shows representative confocal images of sites without ducts acquired by confocal fluorescence microscopy (Fig. 2a-e), nuclei isolated with MSER at those sites (Fig. 2f-j), and sites in the corresponding histologic slide with H&E staining that have similar histology to the confocal sites (Fig. 2k-o). Nuclei were false-colored green and overlaid onto the original images for visualization. As seen, nuclei are isolated at the periphery of adipose cells (Fig. 2f) and are dispersed throughout the fibrous tissue image (Fig. 2g). Denser clusters of nuclei are isolated in and around lobules (Fig. 2h). Nuclei are the most dense at sites with malignant tissue, including IDC and ILC (Fig. 2i, j).

**Fig. 3:**
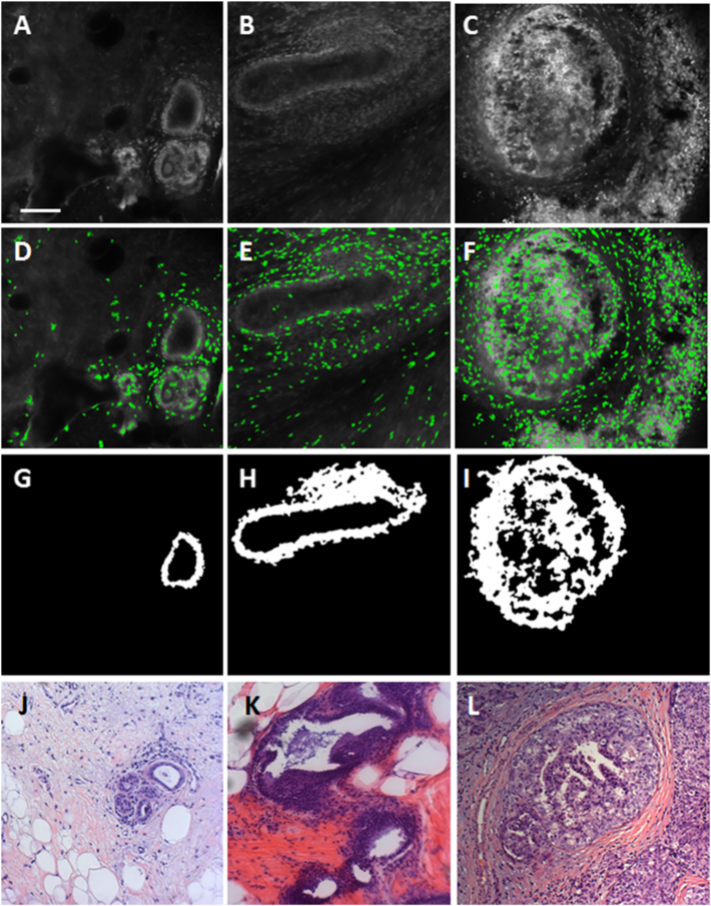
Representative confocal images of normal, non-hyperplastic ducts (**a**), hyperplastic ducts (**b**), and ductal carcinoma in situ (**c**) analyzed with the nuclear segmentation algorithm (middle row) and with the ductal segmentation algorithm (bottom row). **d**-**f**: Nuclei segmented by identifying maximally stable extremal regions (MSER) are false colored green and overlaid onto the raw confocal fluorescence image. **g**-**i**: Breast ducts segmented using the ductal segmentation algorithm. **j**-**l**: Histologic slides with H&E staining show similar histology to confocal images in **a**-**e**. Slides were prepared with the same specimens from which confocal images were acquired. Scale bar is 100 µm

Figure 3 shows representative images of breast ducts acquired with confocal fluorescence microscopy (Fig. 3a-c), nuclei that were isolated at sites with breast ducts using MSER (Fig. 3d-f), ducts that were segmented with the ductal segmentation algorithm (Fig. 3g-i), and sites in the corresponding histologic slide with H&E staining that have similar histology to the confocal sites (Fig. 3j-l). Nuclear density in and around the ducts increases from the non-hyperplastic duct, to the hyperplastic duct, to DCIS (Fig. 3d, e, f). However, relatively few nuclei are successfully isolated using MSER within the non-hyperplastic and hyperplastic ducts, which is most likely due to the fact that the borders of individual nuclei are difficult to visually discern in confocal fluorescence images. The images of sites isolated with the ductal segmentation algorithm show well-defined lumens in both the non-hyperplastic duct and hyperplastic duct (Fig. 3g and h). Conversely, the image of DCIS shows bridges of cells crossing the lumen to create a cribriform pattern with several lumens.

**Fig. 4:**
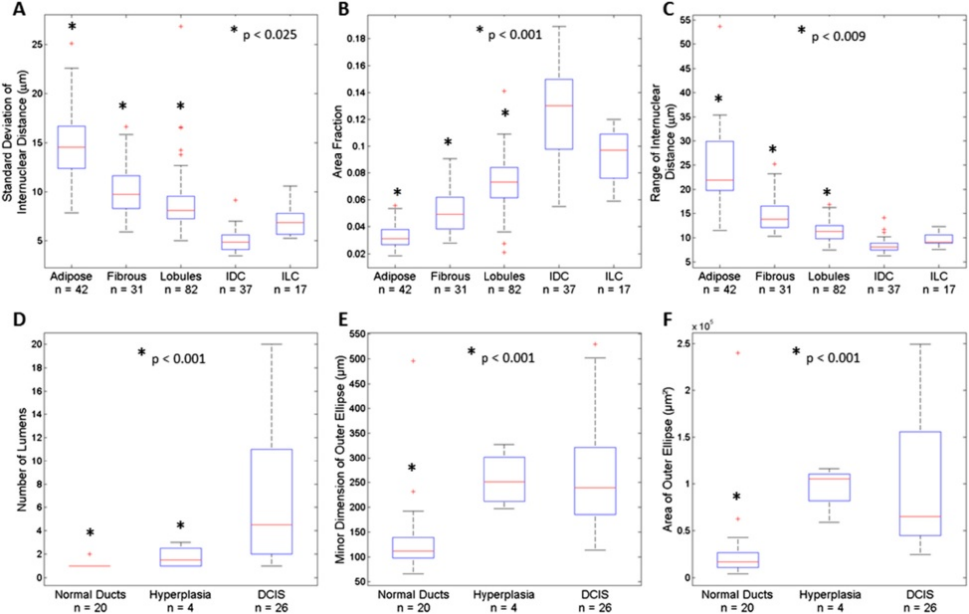
Mean value of parameters used to separate malignant from benign sites. Nuclear parameters calculated with the nuclear segmentation algorithm are shown for all adipose, fibrous, lobules, invasive ductal carcinoma (IDC), and invasive lobular carcinoma (ILC) sites; **a**: standard deviation of inter-nuclear distance; **b**: area fraction; **c**: range of inter-nuclear distance. Ductal parameters calculated with the duct-based segmentation algorithm are shown for all normal, non-hyperplastic ducts, hyperplastic ducts (Hyperplasia), and ductal carcinoma *in situ* (DCIS); **d**: number of lumens; **e**: minor dimension of outer ellipse; **f**: area of outer ellipse. The number of sites represented in each box is represented by *n*. Significant differences between mean values of parameters measured at benign and malignant sites are indicated by asterisks (*)

The parameters that yielded the highest performance for distinguishing between benign and malignant sites are shown in Table 2. We evaluated the performance of nuclear parameters for classification of benign and malignant features in all sites and in subgroups of sites that did or did not contain ducts to determine the groups for which nuclear parameters had the highest classification accuracy. We only evaluated the classification accuracy of ductal parameters at sites that contained ducts. Nuclear parameters measured at non-duct sites achieve higher performance (AUC = 0.93) than nuclear parameter measured at duct sites (AUC = 0.69). Conversely, ductal parameters achieve higher performance (AUC = 0.92) than nuclear parameters for classification of duct sites (AUC = 0.69). These findings suggest that a combination of nuclear parameters measured at non-duct sites and ductal parameters measured at duct sites may yield improved separation between all benign and malignant sites.

**Table 2:** Summary of top performing parameters for distinguishing between benign and malignant sites measured using the nuclear and ductal segmentation algorithms

Group	Performance metric	Standard deviation of inter-nuclear distance	Area fraction	Range of inter-nuclear distance
*A. Classification by nuclear parameter – all sites*	AUC	0.87	0.86	0.87
	Sensitivity	78	76	76
	Specificity	82	79	85
*B. Classification by nuclear parameter – non-duct sites*	AUC	0.93	0.92	0.91
	Sensitivity	85	80	81
	Specificity	88	87	88
*C. Classification by nuclear parameter – duct sites*	AUC	0.68	0.72	0.74
	Sensitivity	46	65	62
	Specificity	100	70	96
Group	Performance metric	Number of lumens	Minor dimension of outer ellipse	Area of outer ellipse
*D. Classification by duct parameter – duct sites*	AUC	0.92	0.83	0.82
	Sensitivity	88	73	81
	Specificity	88	79	75

A: Nuclear parameters measured at all sites using the nuclear segmentation algorithm. B: Nuclear parameters measured at all sites except those with breast ducts (normal ducts, hyperplastic ducts, and ductal carcinoma in situ) using the nuclear segmentation algorithm. C: Nuclear parameters measured at sites with breast ducts using the nuclear segmentation algorithm. D: Ductal parameters measured at sites with breast ducts using the ductal segmentation algorithm. *AUC* area under the curve

Boxplots showing the mean and interquartile range of the top three performing nuclear parameters are shown in Fig. 4a-c. Both StdIND and Range IND decrease from adipose to fibrous to lobules to ILC to IDC (Fig. 4a and c), while AF increases from adipose to fibrous to lobules to ILC to IDC (Fig. 4b). This trend suggests that the number of clusters of nuclei increases from adipose tissue, which has the fewest, to IDC, which has the greatest number of clusters of nuclei. All comparisons between benign (adipose, fibrous, lobules) and malignant (IDC, ILC) sites were significant. Similarly, AF increases from adipose to fibrous to lobules to IDC (Fig. 4b), which suggests increasing nuclear density.

**Fig. 5:**
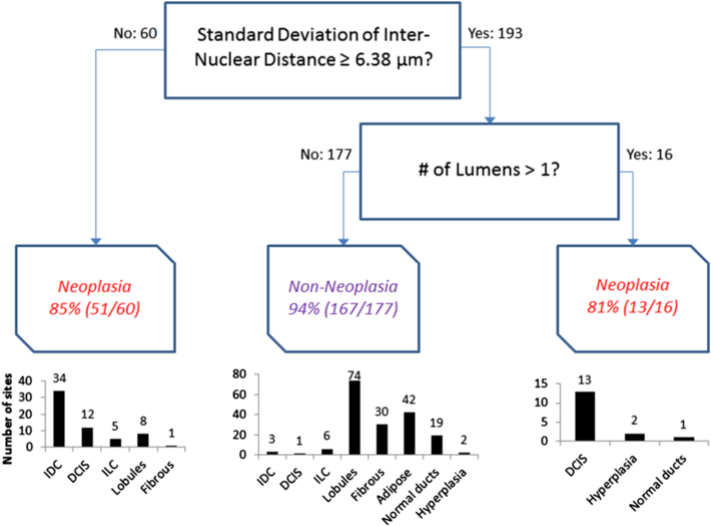
Classification tree automatically generated when all nuclei and duct data was used. Duct- and nuclei-based parameters selected by classification regression tree analysis to optimize separation between benign and malignant sites. Bar graphs show the diagnoses of sites sorted into each classification category

Boxplots showing the mean and interquartile range of the top three performing ductal parameters for duct sites are shown in Fig. 4d-f. DCIS lesions have a significantly higher number of lumens than hyperplastic and non-hyperplastic ducts (*p* <0.001), which is consistent with the cribriform pattern that occurs when abnormally high cellular proliferation causes the luminal space to be filled with epithelial cells (Fig. 4d). Figure 4e shows that the minor dimension of the outer ellipse approximating the duct is significantly smaller in normal, non-hyperplastic ducts than in DCIS lesions (*p* <0.001). There is no significant difference in the minor dimension between ellipses approximating hyperplastic ducts and DCIS lesions (Fig. 4e). Figure 4f shows that the area of the outer ellipse approximating duct area was significantly smaller in normal, non-hyperplastic ducts than in DCIS lesions (*p* <0.001). There is no significant difference between the average area of outer ellipses approximating hyperplastic ducts and DCIS lesions (Fig. 4f).

All 33 nuclear and ductal parameters were used as input for a classification and regression tree (CART) algorithm to automate selection of parameters to discriminate benign and malignant sites. The CART algorithm was pruned to remove redundancies and overfitting to the data set. The classification tree generated through this process is shown in Fig. 5. StdIND with a cutoff value of 6.83 μm is the first decision point selected for classification by the decision tree, followed by number of lumens with a cutoff value of one. StdIND <6.83 μm separates out 52 true-positives composed of IDC, DCIS, and ILC sites and nine false-positives composed of fibrous and lobule sites. The remaining sites enter the second node – number of lumens >1 – which separates out 13 true-positive DCIS sites and three false-positive hyperplasia and normal duct sites. The remaining sites are classified as benign and are composed of 167 true-negative adipose, fibrous, lobule, normal duct, and hyperplasia sites and 15 false-positive IDC, DCIS, and ILC sites. Overall, the model achieved a sensitivity and specificity of 81 % and 93 % respectively, corresponding to an AUC of 0.93 and 90 % overall classification accuracy, as shown in Table 3. If the model is evaluated based on classification of individual histologic types of neoplasia, 92 % of IDC sites and 96 % of DCIS sites were classified correctly. However, the model correctly classified only 35 % of ILC sites. Additionally, leave one out cross-validation was performed, which yielded a cross-validated sensitivity of 75 % and specificity of 93 % (Table 3). Specifically, cross-validation resulted in a 6 % drop in sensitivity (from 81 to 75 %) due to the fact that five additional IDC images were incorrectly classified during cross-validation. When each of these five cases were left out of the original cohort of data used to form the model (in other words during the leave-one-out cross-validation exercise), the cutoff value associated with StdIND dropped. This resulted in each of the five cases being classified as a false negative. For the remainder of the images, the cutoff value associated with StdIND remained the same as it is in Fig. 5, resulting in the same specificity of 93 %.

**Table 3:** Performance of classification tree model for classification of neoplasia, non-neoplasia, and individual histologic types of breast neoplasia in confocal fluorescence images

	Sensitivity	Specificity
Classification tree model	81 % (65/80)	93 % (167/179)
Cross-validated model	75 % (60/80)	93 % (167/179)
	Correctly classified	
All sites	90 % (232/259)	
Ductal carcinoma in situ (DCIS)	96 % (25/26)	
Invasive ductal cancer (IDC)	92 % (34/37)	
Invasive lobular carcinoma (ILC)	35 % (6/17)	

**Fig. 6:**
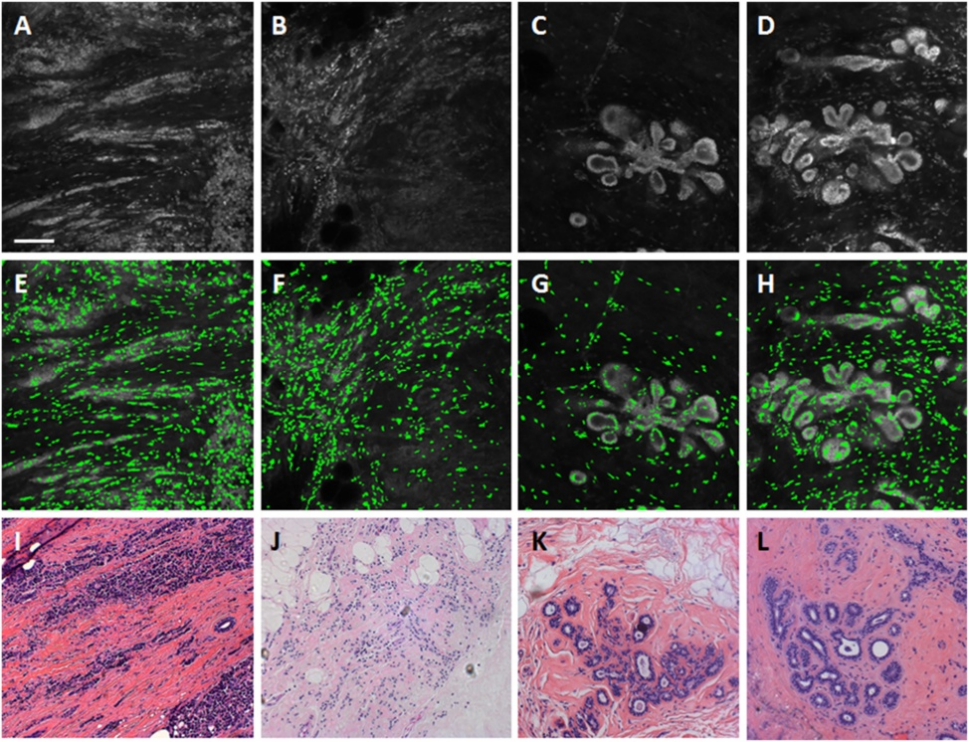
Representative images of sites with lowest classification accuracy in the decision tree model. **a**-**d**: Invasive lobular carcinoma and lobules in confocal fluorescence images. **e**-**h**: Nuclei segmented by identifying maximally stable extremal regions (MSER) are false-colored green and overlaid onto the raw confocal fluorescence image. **i**-**l**: Histologic slides with hematoxylin and eosin (H&E) staining show similar histology to confocal images in (**a**-**e**). Slides were prepared with the same specimens from which confocal images were acquired. **a**, **e**: A true-positive invasive lobular carcinoma (ILC) site; (**b**, **f**): false-negative ILC site; (**c**, **g**): true-negative lobules; and (**d**, **h**): false-positive lobules. Scale bar is 100 μm

As seen in the histograms in Fig. 5, ILC sites account for the largest number of false negatives (n = 11 out of 17 sites) while lobule sites account for the largest number of false positives (n = 8 out of 82 sites). Figure 6 shows representative confocal images of a true-positive ILC, false-negative ILC, true-negative lobules, and false-positive lobules sites (Fig. 6a-d), nuclei isolated with MSER at those sites (Fig. 6e-h), and sites in the corresponding histologic slide with H&E staining that have similar histology to the confocal sites (Fig. 6i-l). As seen, there are large differences in the density and clustering of nuclei between the true-positive ILC site (Fig. 6e) and true-negative lobules site (Fig. 6g). In comparison to Fig. 6e, the false-negative ILC site in Fig. 6f has relatively few nuclei, which appear to be predominately clustered in the upper left region of the image. Conversely, the false-positive lobules site in Fig. 6h contains more nuclei than Fig. 6g, particularly in stromal tissue located in between lobules.

## Discussion

In this study, we performed quantitative analysis of breast histology in confocal fluorescence images by designing algorithms to segment and measure nuclear and ductal parameters. We combined nuclear and ductal parameters to develop a classification tree model to classify malignant from benign changes in the breast parenchyma with 81 % sensitivity and 93 % specificity, which corresponded to an AUC of 0.93 and an overall accuracy of 90 %. The cross-validated model classified the same sites with 75 % sensitivity, 93 % specificity, and 88 % overall accuracy.

Several groups have used automated morphometric evaluation of nuclei in H&E-stained sections of breast tissue [14–17], cytological smears of breast tissue [18], and fluorescence microscopy images of mouse tissue [22, 37] to classify benign and malignant breast features. While these groups demonstrate that quantitative nuclear parameters can be used to classify benign and malignant breast features, some lesions are more difficult to distinguish. For example, Rajesh et al. used automated nuclear morphometry to classify ILC, IDC, and borderline lesions [16]. While significant differences were found between parameters measured for ILC and IDC, no significant difference was found between parameters measured for ILC and benign borderline lesions [16]. We found similar results to the other studies – namely that ILC is difficult to distinguish from non-neoplasia based on nuclear features alone. Additionally, several studies have demonstrated the feasibility of computerized image analysis to distinguish between non-hyperplastic ducts, hyperplastic ducts, and DCIS. Mayr et al. used computerized image analysis to quantify ductal parameters in H&E-stained slides of breast biopsies and found that the most significant parameters for differentiation between normal ducts and DCIS were duct mean diameter and the presence of necrosis [19]. Anderson et al. used a computerized segmentation algorithm to measure parameters of ductal hyperplasia and DCIS in tissue sections stained with the antibody cocktail AE 1/3, and showed that the highest classification accuracy for DCIS was achieved by combining parameters of ducts and lumina [20]. The findings from our work agree with previous studies, which showed that quantitative ductal parameters can be used to classify benign and malignant ducts [19, 20].

The strengths of our study are that we demonstrate that nuclear and ductal parameters can be measured in confocal fluorescence images of clinical samples acquired at the point of care. We perform quantitative analysis of breast tissue architecture without requiring tissue fixation, cutting, and staining and achieve comparable classification accuracy to studies that performed computerized analysis on fixed breast tissue stained with H&E. The model classified IDC and DCIS with greater than 90 % accuracy using parameters that were based on the morphological characteristics of each malignant tissue type. Specifically, IDC was classified with 92 % accuracy using standard deviation in inter-nuclear distance as a parameter, which identifies dense clusters of nuclei. DCIS was classified with 96 % accuracy based on the presence of more than one lumen, which is consistent with the cribriform pattern. Overall we achieve high performance (AUC = 0.93) on a large number of sites (n = 259).

There are several limitations associated with this study. While our data set contains a large number of sites (n = 259), the data were acquired at a single center, and some individual categories, such as ILC contain relatively few sites; therefore, additional work is needed with a large, independent data set including samples from more than one center to validate the reproducibility of these parameters. This is particularly important given the large variance we observed in nuclear parameters. The heterogeneity in nuclear area and distribution within benign breast tissue is a potential source of variance for the nuclear parameters measured in this study. Nuclear area and spacing in breast epithelia vary with a number of conditions, including sexual maturity, pregnancy, menopausal status, use of hormonal contraceptives, as well as the presence of IDC and ILC [1, 2, 40, 41]. Despite this variation, leave-one-out cross-validation of the CART model yields similar performance to the original model suggesting that our algorithm may generalize to an independent data set.

In addition, the algorithm designed for ductal segmentation uses an interactive threshold to convert images from grayscale to binary and a user-defined selection tool to isolate ducts from surrounding nuclei. The ductal segmentation process is a potential source of variability between users, particularly for parameters that could be impacted by a user’s visual assessment of the duct wall boundaries, such as duct wall width. However, the ductal parameter that was ultimately selected for the decision tree model was the number of lumens, which is unlikely to vary at the decision point (number of lumens greater than one) based on slight variations to the threshold value or by excluding surrounding nuclei. This is because it is readily apparent if a duct has one or more lumens based on visual assessment, however, the segmentation algorithm could assist in identifying ducts with more than one lumen. Lastly, examination of the breakdown of false negatives and false positives reveals that our algorithm does most poorly at distinguishing ILC and lobule sites. Specifically 65 % (n = 11 out of 17 sites) of ILC sites and 10 % of lobule sites (n = 8 out of 82 sites) are incorrectly classified. Figure 6 reveals that there are differences in quantity and clustering of nuclei between the true-positive and false-negative ILC sites. In particular, nuclei in the false-negative ILC site appear to be predominately located in the upper left region of the image, suggesting that only the upper left region of the image contains ILC while the remainder of the image may contain other benign tissue. Therefore, the fraction of the image that consists of a malignant tissue type may correlate with the likelihood that it is correctly classified as a true-positive site. Conversely, the false-positive lobule site contains more nuclei than the true-negative lobule site, particularly in stromal tissue located in between lobules. This indicates that the stromal tissue that lobules or other features are embedded within may lead to incorrect classification as a false-positive site. In future studies, additional parameters are needed in order to classify lobules as benign and ILC as malignant with greater accuracy.

It is to be noted that while confocal microscopy provides high-resolution high-quality images, currently its cost, footprint, and maintenance requirements limit the ability to translate this imaging platform to routine usage in patient care. However, this study lays the groundwork for how quantitative analysis could be combined with proflavine staining and fluorescence microscopy. Several applications of fluorescence confocal microscopy can certainly evolve in pathology practice with the potential availability of a user friendly and affordable platform that can be an alternate to currently used modalities for immediate evaluation of fresh tissue. In addition, there could be an opportunity to use a low-cost fluorescence confocal microscope to obtain similar images that may be useful to evaluate fresh tissues in clinical practices with limited resources of professional pathology expertise and laboratory infrastructure to obtain information from the biopsied tissue to guide clinical management [38, 42].

## Conclusions

We measured quantitative nuclear and ductal parameters in confocal fluorescence images of proflavine-stained fresh breast tissue and developed a classification algorithm that distinguished between 259 benign and malignant sites with an accuracy of 88 %. Ultimately, the nuclear and ductal parameters described in this study could be used to develop criteria to automate breast lesion diagnosis for immediate evaluation of fresh tissue at the point of care obviating the need for extensive tissue preparation. Quantitative diagnostic criteria developed on fluorescence confocal images in our study have the potential to enable automated assessment of breast tissue.

## Additional file

Additional file 1: Figure S1. Illustration of methodology used to select MSER intensity parameters. The intensity parameters MaxVariation, MinDiversity, and Delta were selected by varying each parameter one at a time. The area fraction (AF) was calculated for each representative image after each iteration of MSER. Each intensity parameter was plotted as a function of AF. Values for the intensity parameters were selected based on which values correctly isolated nuclei from the representative images and which values led to the largest differences between tumor and benign images. Scale bar 100 μm. (ZIP 140 kb)

## Abbreviations

AF: area fraction
AUC: area under the curve
CART: classification and regression tree
DCIS: ductal carcinoma in situ
H&E: hematoxylin and eosin
IDC: invasive ductal cancer
ILC: invasive lobular carcinoma
IND: inter-nuclear distance
MaxArea: maximum area
MaxVariation: maximum variation
MinArea: minimum area
MinDiversity: minimum diversity
MSER: maximally stable extremal regions
PBS: phosphate-buffered saline
ROC: receiver operator characteristic
StdIND: standard deviation of IND
