# Supplementary figures and images for: Micro-anatomical quantitative optical imaging: toward automated assessment of breast tissues
Source: Breast Cancer Res. 2015 Aug 20;17(1):105. doi: 10.1186/s13058-015-0617-9 (PMC4545917; doi:10.1186/s13058-015-0617-9)

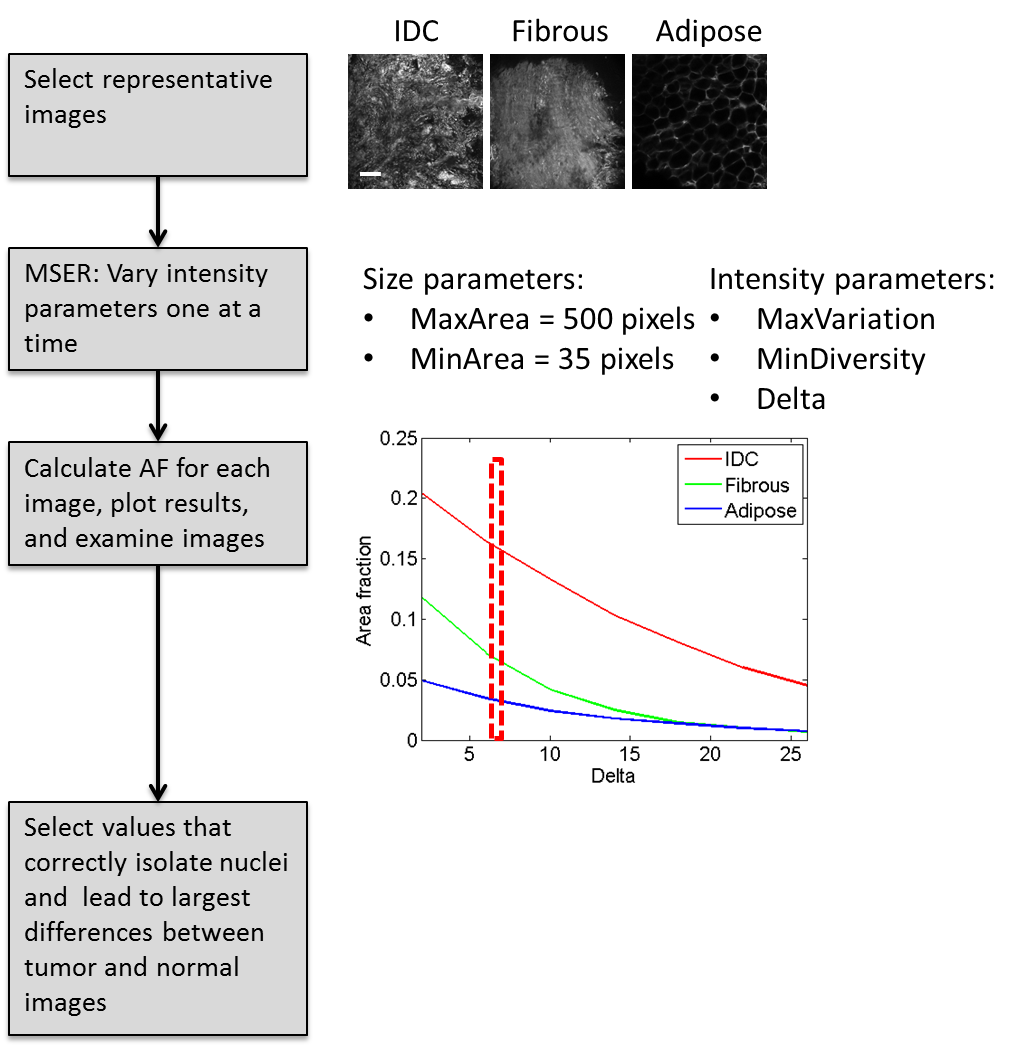

Supplement: Additional file 1: Figure S1. — Illustration of methodology used to select MSER intensity parameters. The intensity parameters MaxVariation, MinDiversity, and Delta were selected by varying each parameter one at a time. The area fraction (AF) was calculated for each representative image after each iteration of MSER. Each intensity parameter was plotted as a function of AF. Values for the intensity parameters were selected based on which values correctly isolated nuclei from the representative images and which values led to the largest differences between tumor and benign images. Scale bar 100 μm. (ZIP 140 kb) [file 13058_2015_617_MOESM1_ESM.zip › 13058_2015_617_MOESM1_ESM.bmp]
